# The Paramount Duty Texas Owes to Her People*Read in the Section of State Medicine at the Thirty-fourth Annual meeting of the State Medical Association of Texas, at Dallas, Texas, May 8, 1902, and by resolution ordered printed in pamphlet form for distribution for “educational purposes.”

**Published:** 1902-06

**Authors:** F. E. Daniel

**Affiliations:** Austin, Texas


### TEXAS MEDICAL JOURNAL.

ESTABLISHED JULY, 1885.

PUBLISHED MONTHLY.—SUBSCRIPTION $1.00 A YEAR.

Vol. XVII. AUSTIN, JUNE, 1902. No. 12.

### The Paramount Duty Texas Owes to Her People.*

BY F. E. DANIEL, M. D., AUSTIN, TEXAS.

[Dr. Daniel prefaced the reading of the following paper by expressing much gratification at hearing Governor Sayers, who had addressed the Convention that day, express himself as deeply interested in the question of protecting the public health, and in favor of a State Board of Health. He regretted, however, that Governor Sayers had not so expressed himself and shown the same interest when the several committees from the State Medical Association called on him prior to the last session of the Legislature and sought his aid and co-operation in securing legislation for the protection of the public health. Dr. Daniel also disclaimed any intention to reflect upon the distinguished gentlemen, who had as effectively as possible administered the very insufficient, defective and bunglesome quarantine laws, criticising the law and not the officers. —Ed.]

Savages or beasts must adapt themselves to their environment or perish. Civilized man conquers his environment. He makes the material resources of his surroundings and the forces of nature subservient to his uses and the advancement of civilization. In the ability to do this lies his chief claim to supremacy in the animal kingdom. The inexorable law of the survival of the fittest in the fierce struggle for existence, applies to races and nations, and is the chief factor in their evolution into strength, stability and permanency. That nation is not the fittest that simply has the largest population, though population is the first requisite to national

*Read in the Section of State Medicine at the Thirty-fourth Annual meeting of the State Medical Association of Texas, at Dallas, Texas, May 8, 1902, and by resolution ordered printed in pamphlet form for distribution for "educational purposes."

strength and expansion. A people may be so numerous, but defective in the elements of vitality, that, like an overburdened fruit tree, where none of the fruit may ripen, it goes to pieces of its own weight; or they stagnate until cholera or plague relieves the congestion, or a stronger, a fitter race, reduces them to subjection. A healthy, strong, vigorous and enlightened population makes a nation great. Hence, the prime consideration is physical health. That State which overlooks this, or ignores a fact so well understood that it has long since become a maxim: "The welfare of the people is the supreme law," commits a fatal error.

The people are the power in a State. They elect one of their number to govern them, and others to represent them and express their will in the legislative halls, and make known their needs. They stand in relation to their governor as children to a father, and look to him for protection, not only in their property rights and the fruits of their labor—both of which are safeguarded in Texas—but in those interests that are a thousandfold dearer to every man, life, health, home and hearth. Socially, they are "citizens," and each contributes his share to the advancement of all that makes for wealth, strength, enlightenment, safety and prosperity, and the governor is the honored head of the socio-political body. In war the citizens are the State's soldiers and defenders, and the "father," the "chief executive," is their commander-in-chief.

That wisdom and promptness would characterize his action were his "children," "fellow citizens," "soldiers" threatened or being devastated by any visible and tangible danger, goes without saying. That both he and the representatives of the people's interests should so long have shut their eyes to the dangers which are as real and as deadly, though intangible, as would be the invasion of an armed host; that they should shut their ears to the warnings of those whose profession and training peculiarly fit them for knowing and appreciating the dangers to the public health by reason of unsanitary conditions, and to the protests of the commercial interests of the State, is as remarkable as it is disgraceful. Such neglect, and the perpetuation of an obsolete and ineffective quarantine instead, is ruining commerce and driving trade from our borders. It is paradoxical. Every safeguard is thrown around property, but no protection is given the people against deadly disease, and against the deadlier danger, the "drugless" doctor! For a quarter of a century the Texas State Medical Association has endeavored to make the Governor and the Legislature see the necessity for protection against diseases other than that afforded by the barbarous and ruinous quarantine, a system that takes cognizance of nothing but diseases of foreign origin, and which, panic-stricken upon the first alarm of yellow fever, locks the wheels of commerce, stops all immigration and traffic, even to refusing entrance to a flat carload of railroad iron! This Association has for twenty years put its finger upon each source of such dangers and said to Governor and Legislature: "See, here it is: polluted water, reeking with typhoid fever, diptheria, dysentery! See, here it is: consumptives by the score, traveling in the deadly draperied and upholstered sleeping cars, and putting up in the stuffy, ill-ventilated and seldom-aired rooms of hotels and boarding houses, thus scattering, broadcast, the seeds of the deadliest and most fatal contagious disease that afflicts mankind. See, here it is: a 'doctor,' an ignoramus, because he is 'drugless,' is exempt from the operation of the only medical law we have ever had in Texas that amounted to a row of pins, and which scarcely amounts to much more. Because this 'doctor' 'gives no medicine' he is at liberty to pull on the fractured arm of a child until the child is 'past praying for,' under the impression, as he expresses it, 'the arm was outer j'int.' See, ten to fifteen per cent. of the blind of the State, whose care, education and keeping cost the people a hundred times as much as it would have cost to prevent it, are made blind by failure of 'doctor' or midwife to recognize infantile sore eyes and properly treat it. See, the dirty midwives spread puerperal sepsis unhindered by law."

It is a shame on the people of Texas, a reproach to the great State, a blot upon our boasted civilization, that such things should be; that a State with 3,000,000 citizens, progressive, intelligent, industrious, loyal, producing about twenty per cent. of the world's supply of the great staple so indispensible to the wants of the millions; a State the extent of whose material resources would baffle the wildest guess; a territory destined some day to be an empire—capable of sustaining a population of half a thousand millions, and in whose vaults lie locked up—dead—a yearly average of $4,000,000, should refuse, fail or neglect to expend a portion of that great surplus in rational efforts to save its people from those diseases originating in our midst that destroy annually more lives than all the cholera, plague, yellow fever, smallpox and war combined.

I quote from an address by Col. A. P. Wooldridge, of Austin, president of the City National Bank, and president of the Texas Bankers' Association: "In February last there was nearly $4,000,000 in the State treasury. Thus we have a grand total in and out of the treasury in the early part of this year of, say, $16,000,000 of actual cash. From these calculations, which will be found to be approximately correct, we find that in the early part of the present year, 1901, probably at this very time, one-fourth of all the cash in Texas is hoarded within its treasury vaults, and this, too, the hard-earned money of the people, taken from them in taxes, and kept not over securely and where it can do no possible good."

That representatives of the people should not demand protection of the health of the people, the fundamental source of wealth, strength and prosperity, is an almost incredible and unpardonable fault. It is their duty to do it. It is the sacred duty, imperative and pressing, incumbent on them and upon the Governor, to see that his "children," his "fellow citizens," his "soldiers," the units of the great whole which make up the great State of Texas, are protected from dangers of which the majority have no conception, and therefore cannot avoid. It is the paramount duty of the hour! Texas, great in all else, should no longer be pointed at with ridicule, because she has never instituted that first essential to a knowledge of the diseases which decimate her population every decade—

a record of vital statistics. Without this knowledge no reform in the way of preventive measures is possible.

This is a vast subject, and I cannot enlarge upon it. Much that pertains to it, while a sealed book to the people and the average lawmaker, is merely elementary knowledge to this body and all enlightened sanitarians. I will only say that the death of every baby, not to say man, is a distinct money loss and a loss of "potential" to the State. The death of every baby or child reduces the average longevity of the people and affects the life expectancy. It is a sad thing to see a baby, or any one, die of any preventable disease, and it is a crime, chargeable against the State, that any should so die. But when we see a strong citizen, a progressive business man, an upbuilder of civilization, stricken down by a miserable typhoid germ which he has unconsciously swallowed in the well water of his home, it may be, or the unfiltered filth of some city drained into the river and distributed by a costly system of waterworks from a reservoir costing $2,000,000 and for which the city (in this case Austin) is bonded, the polluted water from which was once served to soldiers in a camp on its watershed and to citizens, it is doubly sad; it is a shame. To paraphrase Whittier's famous lines, "The saddest words of tongue or pen are these: 'It might have been' "—prevented. Poor consolation to the widows and orphans; to the hosts of attached friends. Is it not the duty of the State, the Governor, the lawmakers, to see that in future all such deaths are prevented? In the name of God and humanity, yes!

This is a subject so prolific of thought and pregnant with importance that I can not in the brief time allotted to reading a paper, do more than touch it. It is a subject upon which I have bestowed much thought, made much observation and by tongue and pen have "harped" it until it has become tedious. We "have piped, but they wouldn't dance." A prophet not without honor save in his own country, (I hope I will be pardoned for saying), was recently illustrated in a small way in my own experience. At the request of the progressive and enlightened editor of *The Current Issue,* a popular magazine published at Austin, I wrote up the subject of vaccination, and showed the necessity of a law that shall make it practically compulsory. The State Board of Health of Florida ordered one thousand pamphlet reprints of the article for educational purposes in that State; none were used in Texas.

I cannot go into the details of the many sanitary evils that should be corrected at once by the State. I can only allude to the necessity of protecting all water supplies from pollution, protection of the people from quack doctors, "osteopathy," "Christian Science," and all such absurdities and abominations; from consumption, by compelling the railroads to sterilize all cars and abolish all upholstered equipment; all hotels to sterilize rooms occupied by consumptives or persons with other contagious disease; to institute drainage, ventilation, regulate plumbing, enforce vaccination, etc. In a word, the pressing necessity is a State Board of Health, composed of the ablest and most experienced sanitarians of the State, and the appointment of such board, which should be paid sufficient salary to enable every member to give his whole time to its important duties, is the paramount duty Texas owes to her people. Details should be left to this Board, the most important of which is the institution at once of a system of vital statistics. It is almost incredible, yet it is a fact, that there is in Texas no record from which it may be proven in court, should it become necessary to do so, that any citizen was ever born in this State or that he ever died. There is no way to find out how many people die yearly in Texas, nor of what they die. I say that this is a reproach that Texas should no longer endure. There is an awakening all over the world to the ravages of the dread white plague, and tuberculosis congresses are being held in America, Europe and Asia to discuss it, and devise means for its arrest. Texas should wake up and take a hand. Texas must admit that smallpox is now permanently endemic in her borders, and she is not able to suppress it. It should bring the blush of shame to our lawmakers to have to admit that while such in the case, Japan, Japan whose people have just emerged from barbarism and who, forty years ago, were fighting with bows and arrows, has exterminated smallpox and has no quarantine against yellow fever; that poor, old, effete Spain has exterminated smallpox; that Germany has exterminated smallpox, all by intelligent legislation—the practical enforcement of vaccination. France has just passed a compulsory vaccination law.

Let us see for a moment how outside people, especially "capital," look at the "sanitary" situation in Texas, and its sanitary needs. Let us see it through the specs of the Merchants' Association of New York, and judge how it affects investments in Texas. It will be remembered that a special committee representing that body of capitalists, by invitation of our Governor and the Legislature, visited Texas about a year ago. They have published their report. During their visit I had several protracted interviews with their sanitary member, Dr. George A. Soper, engineer and chemist, and it was in my power to give him (and I did so with great pleasure) much information that he wanted. Dr. Soper's contribution to the report is embraced under several heads: "Natural Health and Climatic Conditions," Sectional Characteristics," "Temperatures," "Irregularities of Climate," "Health Resorts," "Care of Public Health" (he should have said "neglect of public health"), "State Health Organizations," "Quarantine," "Relation between Sanitary and Commercial Progress," "Conclusions." With your indulgence, I will read some extracts from his report:

"There are many sources of complaint for the want of thoroughly organized central health department in Texas, not the least of which relates to the absence of provision for the collection of vital statistics. No vital statistics are collected.

"In the absence of data regarding the distribution of disease, the healthfulness of the cities and towns is not accurately known.

"Streets, as a rule, are not paved, except in the larger cities. The cleaning of streets and the collection and final disposal of garbage are subjects which have not thus far received the attention they deserve. * * * The free use of disinfectants too often takes the place of the means of preventing diseases which are based upon thorough order and cleanliness."

Of the Texas quarantine, Dr. Soper says: "It is customary for the Governor to issue a proclamation each spring, declaring quarantine against seaports south of twenty-five degrees north latitude, the duration of this embargo usually extending to about six months. To these regulations and to the provisions of the State quarantine law (Title XCII, Revised Civil Statutes), which contains the authority upon which the quarantine regulations are founded, there has been an active protest by the medical professioin and business fraternity for years. The argument is made that the regulations are unnecessarily severe, subject to various interpretations requiring an unauthorized exercise of power for this enforcement, and that they are liable at critical times to fail in accomplishing their purpose. There is no doubt that the quarantine regulations, as often interpreted, are very severe. In this connection, it is interesting to refer by comparison to the action of other Southern States which are also exposed to yellow fever. Realizing the commercial importance of establishing uniform and intelligent regulations for the management and prevention of yellow fever epidemics, all of the Gulf States, with the single exception of Texas, have adopted a code of quarantine rules known under the term of the Atlanta Convention. There is no clear reason why Texas has not availed herself of the benefits thus offered. Not only would such action aid in protecting her citizens, but it would greatly lighten the restrictions now placed upon commerce.

"As to the interpretation which may be put upon the existing quarantine law of Texas, it is only necessary to refer to the frequent misunderstandings which have arisen in its administration. It has been pointed out that the State Health Officer, while nominally in authority, has not been vested with the power of enforcing his directions by legal process. Instances of the arbitrary nature of the power of direction shown during yellow fever scares amply demonstrate his difficulty in overcoming the natural prejudices of people accustomed to the crude method of the shotgun quarantine.

"Under the circumstances it is natural that the numerous accusations of abuse of power should have been made in the medical and public press against the State Health Officer and those who have been called upon to act in concert with him. How far these gentlemen have laid themselves open to such criticism it is not here necessary to say. Their positions have been difficult to occupy to the satisfaction of all interests.

"The cause of sanitation has made slow progress throughout the South, and Texas does not stand alone in failing to take due account and trade-killing absurdities of a totally suspended traffic, quarantined by reason of an excited body of citizens armed with shotguns and rifles, and stampede and panic which occur with the appearance, or a rumor of the appearance, of yellow fever. Nor is the apparent indifference with which modern cities of the South regard advanced methods of sanitation and health administration confined to Texas. It is not intended to condone the quarantine customs and lack of sanitary improvements which, to an impartial eye, are clearly open to criticism, but rather to point out that the practical improvements demanded in her methods of avoiding epidemic diseases must be applied in accordance with natural conditions which are deeply seated and widely prevalent.

"In the industrial awakening which is now taking place, improved sanitary measures are sure to be inaugurated. The losses to business interests, owing to defects in the health laws, by making themselves more and more evident, will in the course of time bring about their own remedy. In a few years such a loss as that of 1898 suffered by railroads, shipping, merchants and others engaged in trade, estimated at from $3,000,000 to $5,000,000 and resulting from a mere scare of yellow fever, will be impossible. * * * The inquiry would be incomplete if it did not point out that the State, by reason of imperfect legislation, and the people by reason of a natural reluctance towards sanitation, have failed to contribute that factor of stability to health conditions which the interests of commerce demands.

"It is clear that a Board of Health is needed in place of the single-headed office now maintained by the State, and that its scope of authority should be commensurate with the great responsibilities which should rightfully come to it. A board, based on principles successfully carried out by such organizations as those of Massachusetts, Michigan and Connecticut, for example, with such changes and modifications as are required to adapt them to the needs of Texas, would be a strong argument to the outside world that the people were laying a strong foundation for their material and permanent advancement. By the operation of a State Board of Health the health conditions in all parts of the great territory could be accurately observed, epidemic diseases most readily controlled, valuable lessons and advice to towns and cities given as to water supplies, sewerage systems, food and drug inspection, disinfection, health ordinances, garbage disposal, etc. Not the least of the duties of the State Board of Health would be the dissemination of a broader and more accurate knowledge of the principles of preventive medicine, so that the people might receive those benefits of the advantages which modern science offers in avoiding communicable diseases. It is not only in Texas that we see the onerous to which they may fairly lay claim as citizens of the great Commonwealth."

I can not better close this brief paper than by a reference to what sanitary science intelligently applied can do and has done toward promoting health, wealth, longevity and consequent strength, permanency and prosperity of a nation; nor need I go far into history for examples. The work in England of Dr. Parr, through whose labors the first public health bill was passed by the British Parliament, a record of vital statistics kept and reform instituted which resulted in the lengthening of the average life of the people, which was truly astonishing, is a matter of history. And the labors of the immortal Howard in the prisons and jails of England mark an era in the history of medicine. It is only necessary to allude to the work done right here at our doors, and only just now completed, by the sanitary department of the United States army in Cuba, under the intelligent and vigorous direction of our brother physician, the great Gen. Leonard Wood, and under the immediate supervision and execution of Surgeon Major W. C. Gorgas, U. S. A.

Dr. Gorgas says that "when the army took charge of the Health Department of Havana deaths were occurring at the rate of 21,252 per year. It gives it up with deaths occurring at the rate of 5,720 per year. It took charge with smallpox epidemic for years. It gives it up with not a single case having occurred in the city for over eighteen months. It took charge with yellow fever epidemic for two centuries, the relentless foe of every foreigner who came within Havana's borders, which he could not escape and from whose attack he well knew that every fourth man must die. It found Havana feared as a thing unclean by all her neighbors of the United States and quarantined against as too dangerous to touch, or even to come near anything that she had touched, to the untold financial loss of both Havana and the United States." At this time, May, 1902, after some two years, when the army turns over the work to Cuban authorities, yellow fever can scarcely be said to exist, and its control is well in hand and its early extermination confidently expected. Quarantine against Havana is no longer necessary.

Let the people draw their own inference of the value of sanitation from this work done.

