# Errata

**Published:** 1902-05

**Authors:** 


### ERRATA.

---

Take lines 36 and 37 from page 451 and read them between lines 44 and 45 on page 450.

For "Parr," read "Farr," on page 451.

